# The magnitude of muscular activation of four canine forelimb muscles in dogs performing two agility-specific tasks

**DOI:** 10.1186/s12917-017-0985-8

**Published:** 2017-03-07

**Authors:** Kimberley L. Cullen, James P. Dickey, Stephen H. M. Brown, Stephanie G. Nykamp, Leah R. Bent, Jeffrey J. Thomason, Noël M. M. Moens

**Affiliations:** 1 Biophysics Interdepartmental Group, University of Guelph, Guelph, ON N1G 2W1 Canada; 2 Institute for Work and Health, 481 University Ave, Toronto, ON M5G 2E9 Canada; 3 School of Kinesiology, Faculty of Health Sciences, Western University, London, ON N6A 5B9 Canada; 4 Department of Human Health and Nutritional Sciences, College of Biological Sciences, University of Guelph, Guelph, ON N1G 2W1 Canada; 5 Department of Clinical Studies, Ontario Veterinary College, University of Guelph, Guelph, ON N1G 2 W1 Canada; 6 Department of Biomedical Sciences, Ontario Veterinary College, University of Guelph, Guelph, ON N1G 2W1 Canada

**Keywords:** Dog, Electromyography, Forelimb muscles, Agility

## Abstract

**Background:**

The purpose of this study was to measure the muscular activation in four forelimb muscles while dogs performed agility tasks (i.e., jumping and A-frame) and to provide insight into potential relationships between level of muscular activation and risk of injury. Muscle activation in eight healthy, client-owned agility dogs was measured using ultrasound-guided fine-wire electromyography of four specific forelimb muscles: Biceps Brachii, Supraspinatus, Infraspinatus, and Triceps Brachii – Long Head, while dogs performed a two jump sequence and while dogs ascended and descended an A-frame obstacle at two different competition heights.

**Results:**

The peak muscle activations during these agility tasks were between 1.7 and 10.6 fold greater than walking. Jumping required higher levels of muscle activation compared to ascending and descending an A-frame, for all muscles of interest. There was no significant difference in muscle activation between the two A-frame heights.

**Conclusions:**

Compared to walking, all of the muscles were activated at high levels during the agility tasks and our findings indicate that jumping is an especially demanding activity for dogs in agility. This information is broadly relevant to understanding the pathophysiology of forelimb injuries related to canine athletic activity.

## Background

Canine agility is a team sport that has grown increasingly popular over the last decade [1, 2]. For example, in 2012 the number of dog entries to sanctioned American Kennel Club agility events was over 1.1 million, at a growth rate of nearly 10% annually over the last ten years [2]. It is a physically demanding sport; a physiological study looking at the hematologic and biochemical changes in dogs participating in agility events found responses consistent with high-intensity anaerobic exercise [3].

As in any sport, there is an inherent risk of injury to the participants. Soft tissue injuries such as strains, sprains and contusions are common in agility; approximately 32% of dog athletes develop an injury [4, 5] and the biomechanical mechanism of injury is often unknown [5]. However, certain activities, such as jumping and climbing the A-frame obstacle, have a higher risk for injury [4, 5]. The shoulder has been identified in two recent retrospective surveys as the most frequently injured anatomical location [4, 5].

With the increasing rates of participation, and the knowledge that nearly one-third of agility dogs experience injuries in the sport, there is also a growing interest in understanding the pathophysiology of shoulder lameness resulting from participation. Canine shoulder injuries are particularly difficult clinical challenges; the soft tissues covering the joint make palpation difficult and the degrees of freedom of movement across the joint complicate diagnosis [6]. Although case reports and surgical techniques are frequently reported, few biomechanical studies describe the normal kinematic, muscular activation or kinetic features of canine gait; a relevant editorial reported that the state of the art for analysis of fundamental biomechanics in canines is decades behind human and equine science [7].

Normal muscle function in healthy canines has been examined at a walk [8–11], trot [11–17], and gallop [11, 18]. Several biomechanical studies have examined clinical canine populations including: partial and pancarpal arthrodesis [19], osteoarthritis [20–22], cranial cruciate ligament rupture [23] and hip dysplasia [24, 25]. However, limited research has evaluated the biomechanics of canines walking on unusual surfacse (such as cross-slopes; [26]), or within canine sport and agility [27–32].

The purpose of this study was to measure the muscular activation in four forelimb muscles while dogs performed agility-specific tasks (i.e., jumping and climbing the A-frame) and to provide insight to potential relationships between level of muscular activation and the risk of injury for each type of obstacle. The four forelimb muscles examined in this study were the Biceps Brachii (BB), Supraspinatus (SP), Infraspinatus (IF), and Triceps Brachii – Long Head (TBLH). These muscles were identifed as being associated with a high risk of injury in the sport [5], and were chosen for further study here both for their importance in canine locomotion [8, 12, 18, 33] and for their role in canines presenting with forelimb lameness in clinical settings [6, 34].

In this study, we were specifically interested in identifying differential changes in the magnitude of forelimb muscle activation when completing the jumping task compared to the A-frame tasks. In addition to examining ascending and descending the A-frame separately, we also compared two current competition heights of the A-frame, where the apex was set at either 1.75 m (high) or 1.67 m (low). Most agility organizations throughout the world have changed their rules governing A-frame apex height to one of these two heights over the last five years [35–38]. This component of the study addresses the ongoing debate within the agility community about the *best* height for dogs to perform the A-frame to reduce the risk of injury.

## Methods

### Participants

Eight healthy, client-owned border collies with a minimum of two years agility experience (ranging from intermediate-level experience at local competitions to internationally ranked competitors) were recruited from the local agility community. The eight dogs (four males, for females) had a mean age of 5.4 ± 1.9 years. The average mass and withers height of the sample were 15.6 ± 2.1 kg and 50.7 ± 1.8 cm. All dogs were evaluated using two independent orthopedic examinations (limb palpation and gait analysis at a walk and at a trot) performed by a board certified veterinary surgeon with experience in kinetic and kinematic gait analysis. All procedures were approved by the University of Guelph’s Animal Care Committee.

### Electromyography (EMG)

The fine-wire electromyography (fEMG) techniques employed in this study are well-established and have been used in neurophysiological and biomechanical studies in humans since the early 1960s [39] and in dogs for the past 30 years [33, 40].

#### Skin preparation

The BB, SP, IF, TBLH muscle bellies were first located by manual palpation. The surface of the skin was prepared for ultrasound with isopropyl alcohol. The skin was anaesthetized using Emla cream (at 1.5 g/10 cm^2^, Astra-Zeneca, Sweden) to eliminate discomfort as the needle penetrated the skin. Bare skin of the inner left thigh was exposed by trimming a 2 cm by 2 cm patch of hair with a clipper; a surface electrode (pre-gelled, Ag-Ag/Cl, 10 mm inner diameter, MediTrace 130, Kendall, MA, USA) was placed here to provide a ground reference.

#### Ultrasonographic examination

We performed B-mode, real-time ultrasonography over the forelimb muscles of interest to guide the needle insertion of the fine wire electrodes (8 MHz micro-convex transducer, GE Healthcare Logiq P5 Ultrasound System) [41]. All insertions were performed by a board certified veterinary radiologist with expertise in musculoskeletal ultrasonography.

#### Electrode insertion

Intramuscular fEMG electrodes (two wires, each 100 μm diameter stainless steel 316 insulated with Formvar; California Fine Wire, Grover Beach, CA, USA) with ~ 5 mm bare ends were inserted into the muscles of interest, on the left side of the dog, using a 27.5 gauge hypodermic needle. The needle was retracted immediately following insertion. Anatomical landmarks were used to determine the needle insertion point and the insertion direction, which have been previously reported [41]. Each needle/EMG electrode was fully sterilized and limited to one use. Electrode wires were connected to the amplifier modules (Trigno Wireless Sensors, Delsys, Boston MA, USA), leaving a loop of excess wire as strain relief, and were secured via a harness (size Small, Ruffwear Web Master™, Oregon USA) to the dog. Dogs were fitted with the harness prior to fEMG electrode insertion. Further details regarding the modifications made to the harness have been presented elsewhere [41].

Videographic data of all trials were collected using a 30 Hz digital video camera (Canon Vixia HFM31). The video records were synchronized with the EMG data using a pulse that was recorded together with the EMG and turned on a light in the video frame. The timing of the "paw down" stride events in the fEMG signals was extracted from the corresponding video records. Preliminary comparisons between 30 Hz and high-speed videeo (420 Hz) identified that the 30 Hz sampling rate was adequate for describing the timing of the gait events (paw down, paw off).

### Procedures

Locomotion data were recorded in a single session for each dog using the following protocol: 1) Baseline measures: three trials were recorded at a walk while the dog covered a back-and-forth pattern of a 6 m distance. These trials were used to allow the dogs to become familiar with the testing apparatus and for real-time assessment of data quality. 2) Ascending and descending the A-frame (2 height conditions): six trials were recorded with the dog performing the A-frame with the apex set at 1.67 m and 1.75 m. The presentation order for performance height was alternated for each participant, although the ascending task always preceded the descending task. The dog started a minimum distance of 4.5 m from the A-frame and began the task by running towards the A-frame when initiated by the handler. After running up the A-frame, the dogs continued over the apex (as per usual agility performance of this obstacle) and exited the A-frame out of the cameras’ field of view. Dogs could continue off the A-frame a minimum of 4.5 m at the end of this task. 3) Jumping task: three trials were recorded with the dog performing two consecutive bar jumps spaced 4.5 m apart set at 55 cm from the ground. The dog started a minimum distance of 4.5 m from the first jump and began the task when initiated by the handler. For all three agility tasks (ascending, descending, and jumping), dogs used a rotary gallop strategy to cover the ground. 4) Repeat of baseline measures: three trials were recorded with the dog repeating the walking task. These post-walking trials were used for EMG normalization (described below), the validation of which has been previously reported [41]. After data collection, the transmitters, harness and fEMG wires were removed and the dogs left the laboratory with their owners.

### Data management and analysis

The EMG signals were amplified (X 909), sampled at 2000 Hz, band-pass filtered between 100-500 Hz with a 2nd order Butterworth filter, rectified and low-pass filtered at 3 Hz with a 2nd order Butterworth filter to remove low frequency movement artifact [42] and high frequency noise. EMG samples for each agility-task stride were amplitude-normalized against the average PEAK amplitude recorded during the three post-experiment walking trials [41]. At least 25 walking strides per dog were used to calculate this value. This normalization technique has been shown to minimize inter-subject variability in human gait analysis [43, 44]. To enable averaging strides across trials, strides were time-normalized to percent of stride (i.e., 100 data points) using a custom-written LabVIEW program (National Instruments, Austin TX, USA).

For the post-walking task (condition used for amplitude normalization), the recorded video was used to identify the timing in the EMG signal that corresponded to “paw down” or the initiation of left forelimb support for each stride until the next consecutive “paw down” event for this limb. For each of the agility-specific tasks, three strides of interest were identified and were generated from EMG signals using a sampling window that began and ended with left forelimb paw contact. These strides were labeled as 1) the pre-transition stride, 2) the transition stride, and 3) the post-transition stride. For the jumping task, the transition stride was defined as the stride where the dog lifted off the ground and jumped over the bar until landing (Jump1_transition and Jump2_transition). For the ascending A-frame tasks, the transition stride was defined as the stride at which the dog lifted off the ground until paw down on the A-frame ramp (Ascend_transition). For the descending A-frame tasks, the transition stride was defined as the stride where the dog left the A-frame and landed on the ground (Descend_transition). The pre- and post-transition strides were defined in all cases as the single strides immediately preceding and following the transition stride respectively (i.e., *condition*_pre, *condition*_post).

All EMG strides were screened by visual inspection and assessed for quality of recording at both the pre-processing and post-processing stages. Recordings that contained high levels of artifact were excluded from analyses as these samples may have led to a false interpretation of muscle activation (111 of 3645 strides were excluded in this way).

### Descriptive and statistical analyses

Temporal and activation level parameters were examined independently for all four muscles sites for the walking task and for the agility-specific tasks, including: peak muscle activity, and duration of stance (% of stride). Linear mixed effect models were used to examine for differences in PEAK EMG amplitude across agility tasks. Each muscle site was examined independently and the models were set with two factors: condition [5 levels: Jump (Jump), Ascend – low apex (Ascend_lo), Ascend – high apex (Ascend_hi), Descend - low apex (Descend_lo), Descend – high apex (Descend_hi)] x stride [3 levels: pre-transition stride (_pre), transition stride (_transition) and post-transition stride (_post)]. Residual and Q-Q plots were examined for each of the four models to assess linearity, homoscedasticity, and the normality of the residuals. For all muscles, the assumptions were supported. When indicated, *post hoc* testing was conducted using least squared means differences. Statistical significance was set at p < 0.05. Based on the visual inspection of results from this analysis, a secondary, exploratory analysis was conducted examining whether there were differences in muscle activity in the jump task between the first and second jump. The models were set with one factor: jump [2 levels: 1. Jump 1 (Jump1_transition), 2. Jump 2 (Jump2_transition)].

## Results

The eight border collies (four males, four females) that participated in this study were all highly trained agility dogs with a minimum of two years competing in agility and a mean age of 5.4 ± 1.9 years. The average mass and height of the sample were 15.6 ± 2.1 kg and 50.7 ± 1.8 cm.

**Fig. 1:**
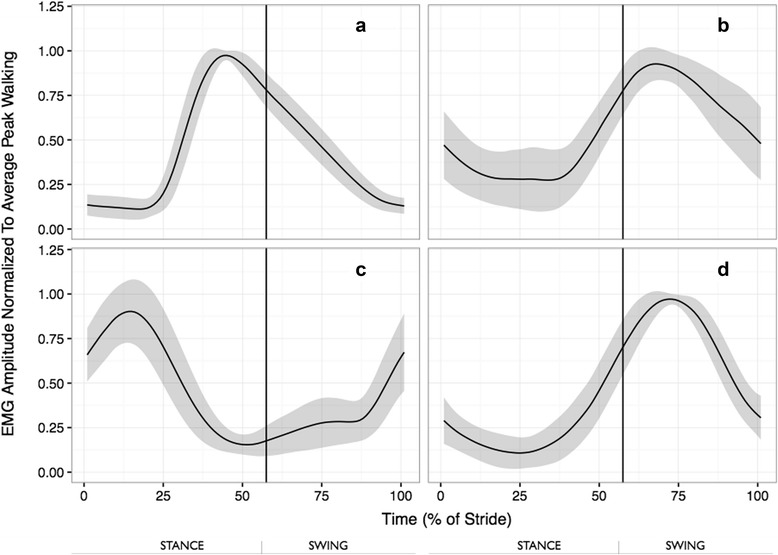
Ensemble-averaged fEMG recordings of all four forelimb muscles observed during the post-experiment walking trials for a representative dog. **a**: TBLH, **b**: BB, **c**: SP, and **d**: IF. The gait cycle is presented in percent of stride, from the initiation of left forelimb floor contact to the subsequent ipsilateral paw strike. The solid line represents the mean activation across the sampling window and the shaded area represents +/- 1 SD across the trials for the given dog’s performance

**Fig. 2:**
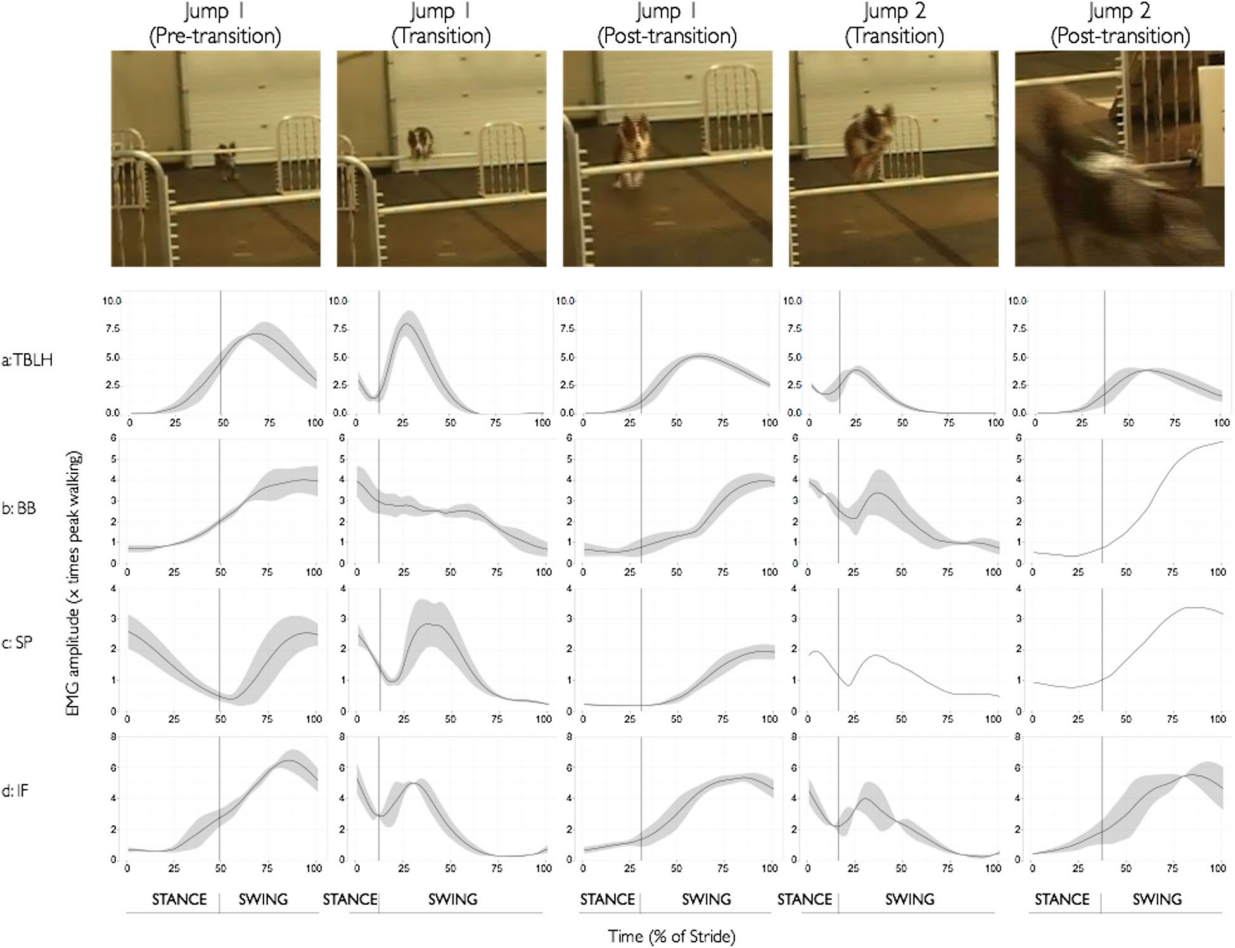
Ensemble-averaged fEMG recordings of all four forelimb muscles for the 1st jump pre-transition strides (Jump1_pre), transition strides (Jump1_transition) and post-transition strides (Jump1_post) and the 2nd jump transition strides (Jump2_transition) and post-transition strides (Jump2_post) during the jumping task for a representative dog. **a**: TBLH, **b**: BB, **c**: SP, and **d**: IF. The still frame images at the top of the figure provide a visual snapshot of each stride in the series for the jumping task. The solid line represents the mean activation across the sampling window and the shaded area represents +/- 1 SD across the trials for the given dog’s performance. When no shaded line appears, it indicates that there was only one performance trial available for this stride

**Fig. 3:**
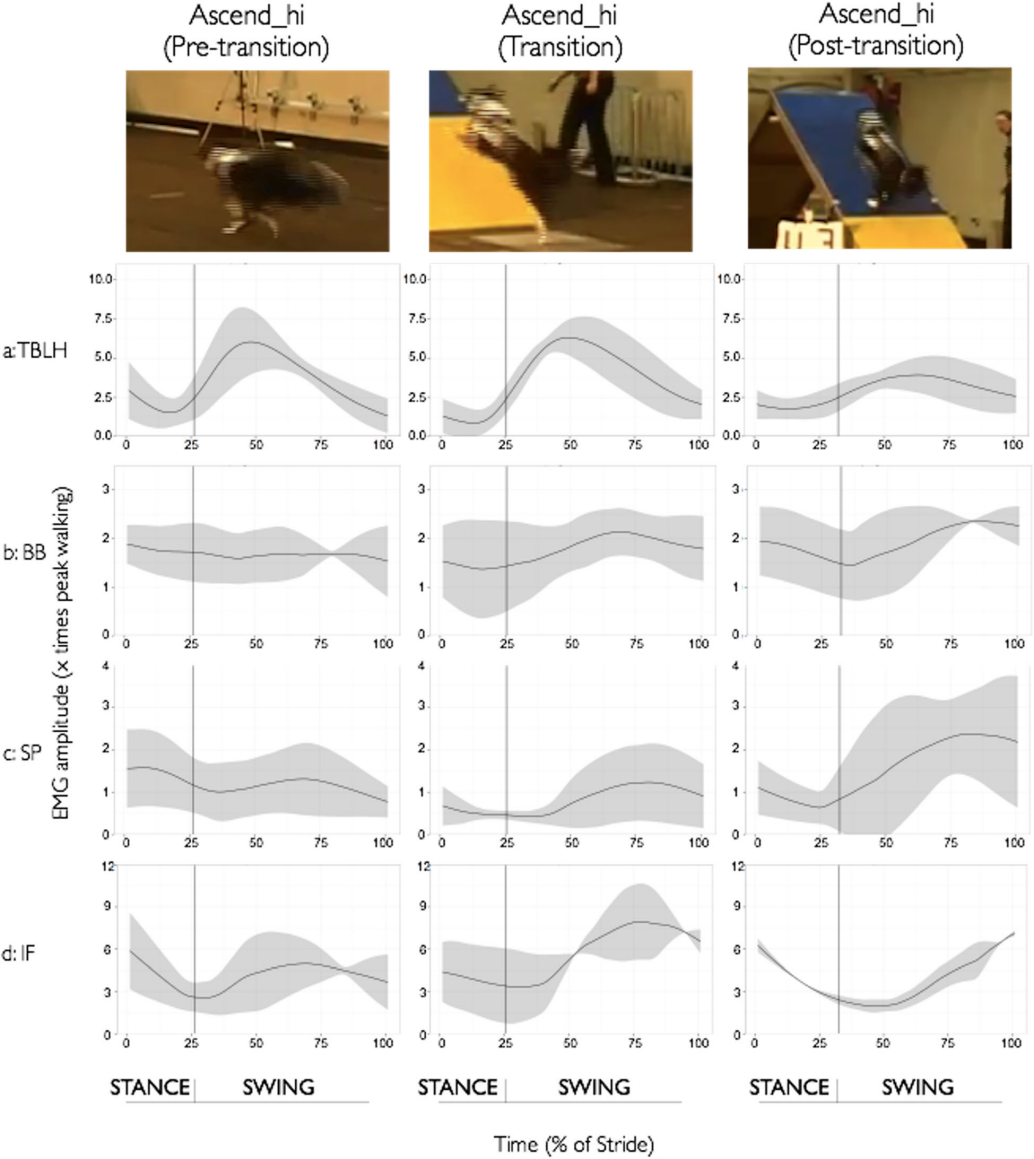
Ensemble-averaged fEMG recordings of all four forelimb muscles for the pre-transition strides (Ascend_hi_pre), transition strides (Ascend_hi_transition) and post-transition strides (Ascend_hi_post) during the Ascending A-frame – High Apex height task for a representative dog. **a**: TBLH, **b**: BB, **c**: SP, and **d**: IF. The still frame images at the top of the figure provide a visual snapshot of each stride in the series for the ascending A-frame task. The solid line represents the mean activation across the sampling window and the shaded area represents +/- 1 SD across the trials for the given dog’s performance

**Fig. 4:**
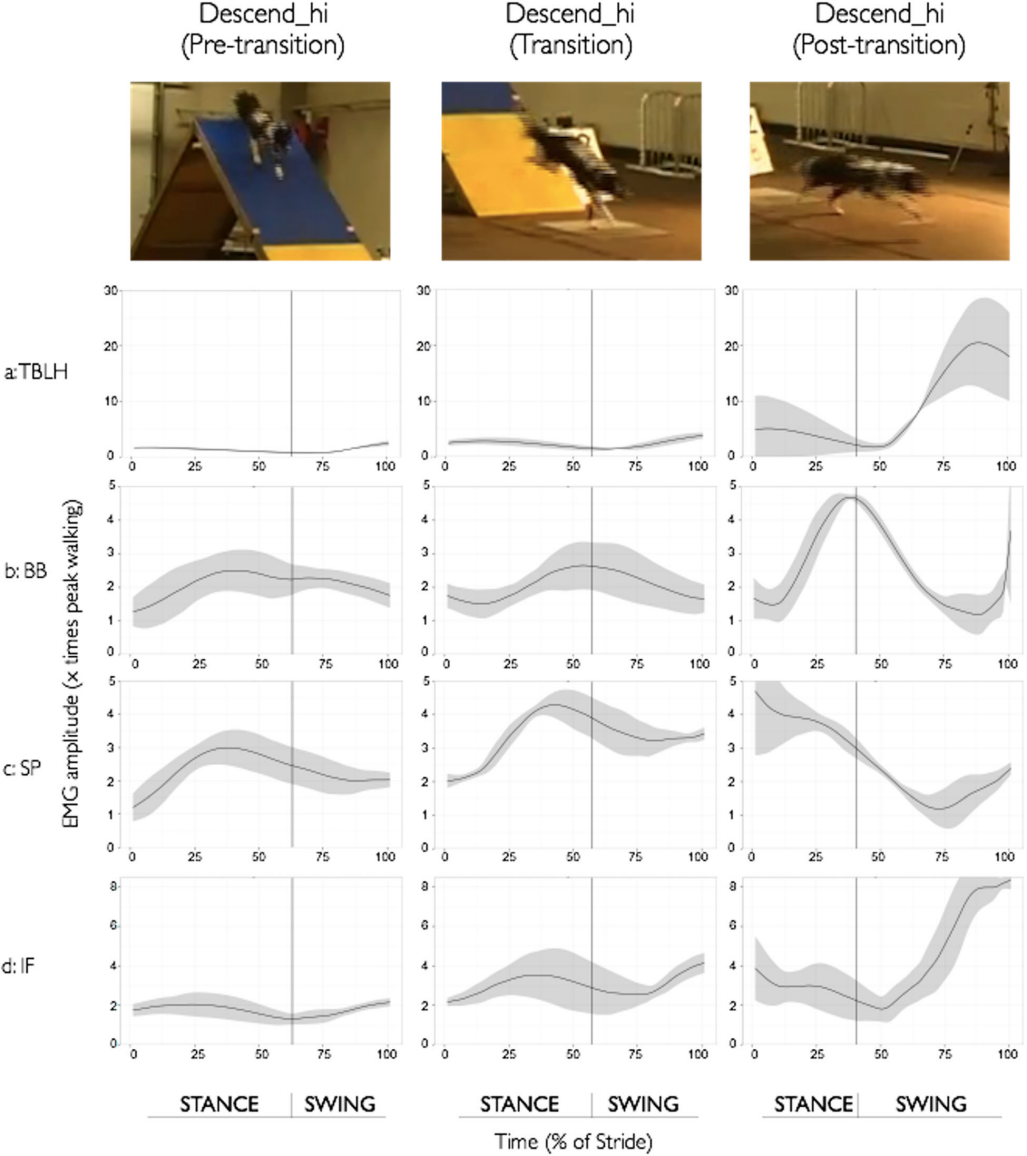
Ensemble-averaged fEMG recordings of all four forelimb muscles for the pre-transition strides (Descend_hi_pre), transition strides (Descend_hi_transition) and post-transition strides (Descend_hi_post) during the Descending A-frame – High apex height task for a representative dog. **a**: TBLH, **b**: BB, **c**: SP, and **d**: IF. The still frame images at the top of the figure provide a visual snapshot of each stride in the series for the descending A-frame task. The solid line represents the mean activation across the sampling window and the shaded area represents +/- 1 SD across the trials for the given dog’s performance

Data for representative participants (Figs. 1, 2, 3 and 4) and sample mean peak activation amplitudes (Table 1) illustrate typical muscle activation patterns during the walking and the agility-specific tasks. Across all agility tasks, for many strides, the four forelimb muscles demonstrated their peak activation levels during the swing phase of the gait cycle (See Figs. 2, 3 and 4).

**Table 1:** Mean peak muscle activation for the TBLH, BB, SP, and IF muscles for the pre-transition, transition and post-transition stride for each agility-specific task (Descend_hi, Descend_lo, Jump, Ascend_hi, Ascend_lo)

Mean peak amplitude^a^ (SD)	Agility-specific task														
	Descend_hi			Descend_lo			Jump			Ascend_hi			Ascend_lo		
	Pre	Transition	Post	Pre	Transition	Post	Pre	Transition	Post	Pre	Transition	Post	Pre	Transition	Post
TBLH	3.1 (1.3)	4.7 (2.2)	7.6 (6.1)	2.9 (1.1)	4.0 (1.6)	6.2 (4.9)	6.0 (3.1)	10.6 (8.5)	4.7 (3.3)	6.8 (2.1)	6.7 (2.7)	6.3 (2.7)	7.4 (2.8)	6.5 (2.9)	6.7 (3.1)
BB	3.1 (1.6)	4.1 (1.9)	4.6 (1.8)	3.1 (1.5)	3.6 (1.5)	4.8 (2.9)	4.3 (3.3)	5.5 (2.9)	2.7 (1.4)	4.4 (2.5)	5.1 (2.8)	4.5 (2.6)	4.0 (2.7)	4.1 (2.5)	3.8 (2.5)
SP	3.1 (1.7)	5.0 (3.0)	5.8 (2.5)	3.6 (1.9)	5.0 (2.5)	5.6 (2.8)	3.3 (1.7)	5.6 (2.6)	2.8 (1.1)	3.6 (1.9)	3.6 (2.0)	3.8 (1.8)	2.8 (2.1)	3.7 (2.6)	3.6 (2.3)
IF	1.7 (0.8)	5.2 (4.2)	6.0 (3.7)	2.6 (1.3)	4.3 (2.7)	5.1 (4.2)	3.7 (4.0)	6.4 (1.9)	4.6 (3.3)	6.7 (2.1)	7.3 (2.3)	6.7 (2.5)	7.4 (2.9)	7.3 (4.3)	7.2 (4.8)

^a^ Peak amplitudes reported in this table have been normalized to the walking trials and should be interpreted as being x *times* that of the peak amplitude observed in the walking trials

Data are presented for each task by stride (Pre-transition, transition and post-transition strides)

In the jumping task (see Table 1 and Fig. 2), the peak activation across all four muscles was substantially greater than that observed during the baseline walking task, ranging from 2.7 times walking (BB Jump1_post) to more than 10.6 times walking (TBLH Jump1_transition). The transition from stance to swing occurred early in the stride for all strides in the sequence (transition strides: 12% for Jump1_transition, 16% for Jump2_transition; post-transition strides: 31% for Jump1_post, 36% for Jump2_post). Across all four muscles, peak activation occurred during the swing phase, except for the transition strides (Jump1_transition & Jump2_transition), where all muscles demonstrated two peaks, one at early stance and a second during mid-swing.

Similar to the jumping task, the peak activations across all four muscles were substantially greater than that observed during the baseline walking task in both of the ascending A-frame tasks (See Table 1 and Fig. 3 (high apex, *low apex is not shown*)), ranging from 2.8 times walking (SP Ascend_lo_pre) to more than 7.4 times walking (IF Ascend_lo_pre). In both tasks involving ascending the A-frame, the transition from stance to swing occurred early in the stride for all strides and was virtually identical in timing between the high and low apex A-frame heights (pre-transition: 26% Ascend_hi and 25% Ascend_lo; transition: 25% Ascend_hi and 25% Ascend_lo; and post-transition: 32% Ascend_hi and 33% Ascend_lo). Across all four muscles, peak activation occurred in the swing phase of the strides.

The peak activations across all four muscles continued to be higher than that observed during the baseline walking task in both of the descending A-frame tasks (See Table 1 and Fig. 4 (high apex, *low apex is not shown*)), ranging from 1.7 times walking (TBLH Descend_hi_post) to more than 7.6 times walking (IF Descend_lo_pre). In both tasks involving descending the A-frame, a greater proportion of the stride was spent in stance for all strides as compared to the jumping and ascending A-frame tasks (pre-transition: 63% Descend_hi and 66% Descend_lo; transition: 57% Descend_hi and 59% Descend_lo; and post-transition: 41% Descend_hi and 37% Descend_lo). Also, in contrast to the other agility tasks, peak activation occurred in stance for many strides for the descending A-frame tasks.

**Fig. 5:**
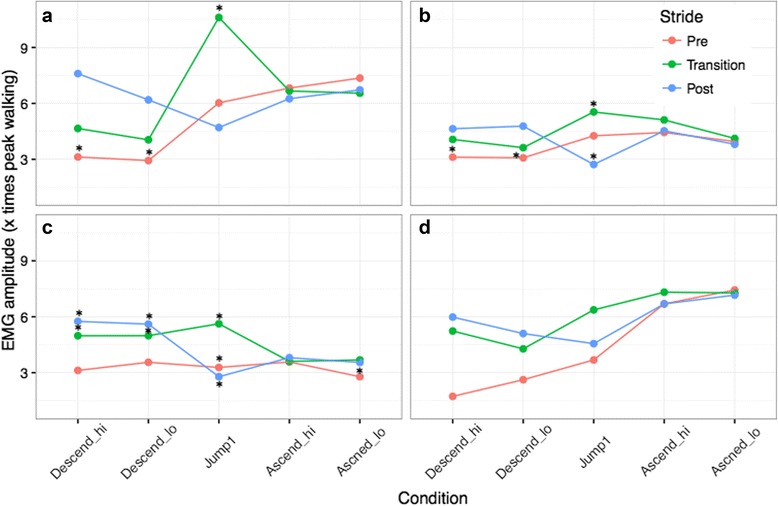
Interaction plots for the comparison of the average Peak EMG amplitudes for each of the agility specific tasks (Descend_hi, Descend_lo, Jump, Ascend_hi, Ascend_lo) by stride type (pre-transition stride, _pre; transition stride, _transition; and post-transition stride, _post) for all four muscles. **a**: TBLH, **b**: BB, **c**: SP, and **d**: IF. * indicates significant differences identified through post hoc analyses

The interaction plots for agility-specific task by stride are presented in Fig. 5 for each muscle site. There was a significant interaction between condition and stride for the TBLH, BB and SP muscles (TBLH & BB: *p* < 0.0001, SP: *p* = 0.009) but not for the IF muscle (*p* = 0.2), although the main effect for condition was trending towards significance for the IF muscle (*p* = 0.055).

Post hoc tests revealed that compared to each of the other conditions, activations for both the TBLH and BB muscles were significantly higher while jumping (Jump1_transition, see Fig. 5a-b, *p* < 0.05), TBLH activation was signficantly lower while descending an A-frame during the pre-transition stride (Descend_pre, see Fig. 5a, *p* < 0.05), regardless of the A-frame height. Activation for the BB muscle was significantly lower than other conditions while landing from a jump (Jump1_post, see Fig. 5b, *p* < 0.05). It was also signficantly low when descending an A-frame during the pre-transition stride (Descend_pre, *p* < 0.05), regardless of the A-frame height.

In contrast, SP activation was highest while leaving an A-frame, regardless of the A-frame height, (Descend_hi_post, Descend_lo_post, see Fig. 5c, *p* < 0.05), followed by the transition strides when descending an A-frame and when jumping (Descend_hi_transition, Descend_lo_transition, Jump1_transition, *p* < 0.05). SP activation was lowest while preparing to take-off and land from a jump (Jump1_pre and Jump1_post, *p* < 0.05), followed by ascending the A-frame (Ascend_hi, Ascend_lo for all strides, *p* < 0.05).

Although not significant, inspection of the means for the IF muscle showed that ascending the A-frame (all strides) required the highest level of muscular activation. The descending pre-transition strides continued to have the least activation in this muscle, consistent with the other muscles (see Fig. 5d).

Across all four muscles, there were no significant differences observed in peak muscle activation required to ascend or descend the A-frame between the low and high apex conditions for the pre-transition, transition and post-transition strides (See Fig. 5a-d).

Visual inspection of the transition strides for Jump1_transition and Jump2_transition, revealed what appeared to be a pattern; several dogs had a higher peak amplitude in the first jump compared to the second jump (See Fig. 2). When examined statistically, this difference was not significant (TBLH: *p* = 0.14, BB: *p* = 0.22, SP: *p* = 0.14, IF: *p* = 0.27). Dogs were able to self-select how many pre-jump strides they took before taking off. We examined the results to explore whether the number of pre-jump strides influenced the muscle activations in more detail. There did appear to be a difference in peak muscle activity when taking into account the number of pre-strides taken before lift-off; however there were not enough data points to run a statistical analysis (data not shown)

## Discussion

This study described the activation patterns of four forelimb muscles for highly trained agility dogs completing two agility-specific tasks: jumping, and ascending/descending the A-frame. The results have provided the first recordings of muscle activation for these agility tasks and the first in vivo recordings of these muscles in dogs using a minimally invasive, ultrasound-guided fEMG insertion technique. This confirmation via ultrasound, and minimal level of invasiveness, are important elements as previous studies in dogs either used blind insertion of the fine-wire electrodes [8, 12, 18], or employed a highly invasive surgical implantation technique [11, 14, 15, 17, 33].

Across each of the agility-specific tasks, the magnitudes of the peak activations for all four forelimb muscles were consistently high relative to walking. For example, the TBLH demonstrated peak activations during the agility tasks between 3 and 10 times that observed during walking. A similar pattern was observed for the other three muscles (BB, SP, and IF), although the range in activations was slightly smaller (ranging from 3 to 6 (BB & SP) or 7 (IF) times the peak activation observed during walking). During the walking trials, the pattern of activation for the four forelimb muscles were consistent with previous studies [8, 33]. The pattern of activation for the shoulder flexor (TBLH), shoulder stabilizers (SP, IF), shoulder extensors (BB, SP), elbow flexor (BB) and elbow extensor (TBLH) were consistent with expectations based on their function and anatomical locations [8, 12, 18, 33].

Recent work examining the mechanism of human hamstring injuries in over-ground sprinting have demonstrated there is a substantial potential for injury in this powerful extensor muscle during terminal swing [45–47]. Using whole-body kinematics, ground reaction forces and EMG recordings, researchers have determined that hamstring muscles are contracting eccentrically during the late swing phase of over-ground sprinting [45–47] and the maximum activations of the hamstring muscle occurred during terminal swing [46, 47]. Eccentric contractions are known to contribute to injury [48], and have been associated with significant declines in maximum force output as well as histological and structural evidence of damage [49]. Accordingly, as we often observed peak activations in terminal stance and early swing, it is possible that this mechanism of eccentric muscle injury may be responsible for the high incidences of muscle strain injuries in agility [5]; however additional kinematic studies are necessary to test this theory further.

In contrast to the jumping and ascending tasks, the stance time was longer than swing time for the two descending tasks. Additionally, peak activation for the shoulder extensor (BB, SP) and stabilizer (SP, IF) muscles occurred during stance for these strides. This change in muscle activation is consistent with the observation that the forelimbs exert stronger braking forces during downhill grades to facilitate anterior-posterior balance [50, 51], and somewhat related to the observation that down-slope limbs exert greater vertical forces during cross-slope walking [26].

In this study, we used a submaximal dynamic task (i.e., walking) as a reference activity for EMG normalization. Although there is debate in the literature regarding the best normalization technique to use under similar conditions (i.e., when it is impractical to acquire muscle activations from a reference maximal voluntary contraction), this technique has been used successfully to allow direct comparisons between subjects and within-subjects across tasks and testing dates [52–56]. However, one limitation of this technique is that it is difficult to identify the muscle activations relative to the muscles’ maximal capacity, or to discern the relative contributions among the different muscles during these agility-specific tasks. From our previous work, we have learned that the shoulder is commonly injured, especially when jumping and performing the A-frame obstacle [5]. With this study, we have been able to shed light on the relative magnitude of activation across four forelimb muscles when performing agility-specific tasks, several of which are commonly injured in this population [6, 34]. We observed high levels of activation, and timing of peak muscle activation, that are consistent with these injuries.

Peak activations for the both the BB and TBLH occurred in swing during the jumping transition stride. A recent study examining canine joint angles during a similar jump task, found significantly greater flexion in the shoulder and elbow joints during the takeoff phase that carried over into the bascule phase (arc) of jumping [32]. Similar kinematic patterns occur during maximal movement initiation in greyhounds [30]. These phases represent the transition stride in our study. Their findings of greater flexion in these joints are consistent with the high levels of muscle activation we found in the BB (acting as an elbow flexor) and TPLH (acting as a shoulder flexor and elbow extensor) during the transition stride. This activation may represent the stretch-shorten cycle that is associated with storage and recovery of energy during balistic movements [57, 58].

In light of these findings, future work could utilize both whole-body kinematic parameters and ground reaction forces, in addition to EMG measures, to build linked-segment models to examine the dynamics of these forelimb muscles during these specific agility tasks. Attention could be given to the differences in peak activation levels between stance and swing phases in relation to the timing of eccentric and concentric contractions of these muscles. These analyses would be especially helpful in refining our understanding of the nature of the biarticular muscles acting at the shoulder joint during these highly dynamic tasks [59, 60]. Functionally, the shoulder is extending and the elbow is first flexing to clear the jump and then these joint motions are reversed to prepare for landing [32]. Accordingly, the TBLH and BB are active during both stretch and shortening contraction cycles of the muscle. Augmenting the current study’s contribution about muscle activation with information about whether the muscle contractions are concentric or eccentric would further our understanding of the specific etiologies of injuries related to jumping and climbing the A-frame in agility, and help identify which forelimb muscles are at a greater risk for injury within the sport.

Across all agility-specific tasks, there appears to be the most consistency in muscle activation patterns (i.e., least amount of stride to stride variability) when ascending the A-frame, regardless of height. Within the descending the A-frame task, the post-transition stride (Descend_post) always required the highest amount of activation among all four muscles. Although only trending to significance, for the IF muscle, ascending the A-frame was as demanding as the jump condition.

Another interesting finding was the consistency in muscle activation patterns between the two A-frame competition heights tested in this study. Within the agility community, there is much discussion about whether there is an increased risk of injury for the dogs when performing an A-frame set at the higher of these two heights. While our results can not speak fully to the risk of injury since we have not considered joint and muscle forces and moments while performing these tasks, we have clearly documented that there is no appreciable difference between these two competition heights in regards to the levels of muscular exertion in these four specific muscles.

Our previous work has indicated that shoulders are commonly injured when jumping or performing the A-frame task [5]. Based on the muscle activation findings from this study, it is clear that the jump task (and more specifically, the transition stride where the dog lifts off the ground to clear the jump and reaches forward with the forelimb to land) is consistently the most demanding across all four forelimb muscles. In regards to the A-frame task, ascending the A-frame is consistently more demanding for the dog across all muscles than descending the A-frame, with the exception of the final descent stride (i.e., the post-transition stride) after the forelimbs have already touched down on level ground. These findings are consistent with the smaller shear and normal impulses observed during ramp descents compared to ascents [51].

Dogs are typically required to perform many more jumps on any given agility course or training session, compared to completing the A-frame obstacle. Our examination of typical courses designed by judges throughout Europe and North America in 2011 revealed, that on a standard agility course, more than 65% of the obstacles performed in the sequence were jumps. The A-frame obstacle represented less than 1% of the total number of obstacles within the sequence [5]. This greater exposure to the jumps, coupled with our finding of higher forelimb muscle activation requirements during the jumping transition stride, is troubling. Taken together, these findings suggest that dogs repeatedly experience high demand in these forelimb muscles several times in rapid succession when completing a full agility sequence; a sequence, which typically takes between 30-40 s for the fastest dogs to complete. However, in between the performance of individual jump obstacles, the dogs are running on the ground and may be performing different obstacles – activities that likely require lower levels of muscle activation to perform. In contrast, completing the sole A-frame obstacle in the sequence requires less overall muscular activation, compared to jumping, but the dog performs several consecutive strides at this level of activation. This occurs because the ascending and descending tasks do not occur in isolation – i.e., the dog must do both in order to complete the obstacle. Our findings suggest that the level of muscular activation needed to fully ascend and descend the A-frame is similar across each of the six to ten^1^ consecutive strides required to complete this obstacle. Our findings are consistent with the similar magnitudes of shear and normal forces during trotting up and down ramps [51].

The differences in the performance requirements and the muscle activation patterns observed between the jump and A-frame obstacles, suggest that it is likely that the exact mechanism of injury attributable to these obstacles could also be different. For example, these injuries may result from differential magnitudes of risk due to overuse [61] vs. overload [62] mechanisms.

There was a large proportion of the IF muscle fEMG recordings which were excluded from analyses due to poor signal quality (data not shown). It is possible that the harness interfered with this specific muscle since the insertion site for the IF rested underneath the harness structure that the secured the fEMG wires. The other muscle insertion sites were adjacent to the harness rather than underneath it. Future studies should consider alternate harness arrangements that would secure the EMG wires without interfering with the signal quality due to contact.

This study examined muscle activation patterns in four forelimb muscles during two highly dynamic agility-specific tasks. Shoulder injuries are known to be associated with performance of these tasks [4, 5], however, the results from this study, while insightful, only offer one piece of the puzzle in understanding the mechanisms of injury related to these tasks. Future work investigating the kinematics and ground reaction forces during these strides, and more specifically building link-segment models using these measures, is necessary to help shed light on the injury mechanisms related to these agility tasks. Researchers in the UK [27] examined ground reaction forces in dogs performing different jumping tasks and observed high peak vertical forces in the forelimbs (4.5 times body weight) when performing a similar jumping task to the one conducted in our experiment. In their study, dogs jumped 10 cm higher than in our protocol, representing a difference in typical height jumped between agility competitions in these two jurisdictions. To date, there have been no studies reporting on the kinetics related to the A-frame task. Sophisticated computer models have been developed to predict hind limb loading during walking and trotting [63, 64], and two-dimensional (2D) models have been developed for the pectoral limb during walking [65], but these approaches have not yet been applied to agility-specific activities.

In this study, all measures were recorded on the dogs’ left forelimbs. Dogs were not constrained into a specific choice for leading and trailing limbs. It is possible that handness may confound these results, since these tasks require asymmetrical gaits. Dogs were also free to choose the preferred gait between recorded strides of interest. In most cases, and as is expected at high rates of speed, the dogs in this study exhibited a rotary gallop when performing the A-frame and jumping tasks [66]. It is unlikely that this would have a large impact on the findings from this study since these factors would affect both the right and left sides, and our measures were only from the left side. Future studies that specifically manipulate these constraints could shed further light on this matter.

This study successfully described the activation patterns of four forelimb muscles for highly trained agility dogs completing highly dynamic activities. The results have provided the first in vivo recordings of these muscles in dogs using a minimally invasive, ultrasound-guided fEMG insertion technique [41]. The use of intramuscular electrodes is preferred as the problems of electrode movement relative to the muscle and cross-talk from adjacent muscles are minimized when the use of surface electrodes is avoided [42, 67, 68].

## Conclusions

Our findings from the examination of highly trained dogs completing two specific agility-related tasks indicate that jumping is an especially demanding activity for dogs in this sport. Compared to ascending and descending an A-frame, jumping requires the highest level of forelimb muscle activation for all muscles of interest. We also determined that, at least in terms of the levels of muscle activation required to perform them, there was no difference between the two most common A-frame heights used in competitions. Future work should build on these findings to help shed light on the mechanisms related to shoulder injuries associated with these specific agility tasks.

## Abbreviations

BB: Biceps Brachii
EMG: Electromyography
fEMG: Fine-wire electromyography
IF: Infraspinatus
SP: Supraspinatus
TBLH: Triceps Brachii – Long Head

## Footnotes

1 Data were not presented for every stride required to complete the A-frame obstacle. On average, the dog took four strides to complete the ascending task and three to six strides to complete the descending task depending on whether the dog had been trained to run vs. stop at the end of the A-frame.
